# Recent Advances in Cardiovascular Diseases Research Using Animal Models and PET Radioisotope Tracers

**DOI:** 10.3390/ijms24010353

**Published:** 2022-12-26

**Authors:** Weronika Wargocka-Matuszewska, Witold Uhrynowski, Natalia Rozwadowska, Zbigniew Rogulski

**Affiliations:** 1 Faculty of Chemistry, University of Warsaw, Pasteura 1, 02-093 Warszawa, Poland; 2 Institute of Human Genetics, Polish Academy of Sciences, Strzeszynska 32, 60-479 Poznan, Poland

**Keywords:** positron emission tomography (PET), cardiovascular diseases (CVD), molecular imaging, radioisotope marker, animal model

## Abstract

Cardiovascular diseases (CVD) is a collective term describing a range of conditions that affect the heart and blood vessels. Due to the varied nature of the disorders, distinguishing between their causes and monitoring their progress is crucial for finding an effective treatment. Molecular imaging enables non-invasive visualisation and quantification of biological pathways, even at the molecular and subcellular levels, what is essential for understanding the causes and development of CVD. Positron emission tomography imaging is so far recognized as the best method for in vivo studies of the CVD related phenomena. The imaging is based on the use of radioisotope-labelled markers, which have been successfully used in both pre-clinical research and clinical studies. Current research on CVD with the use of such radioconjugates constantly increases our knowledge and understanding of the causes, and brings us closer to effective monitoring and treatment. This review outlines recent advances in the use of the so-far available radioisotope markers in the research on cardiovascular diseases in rodent models, points out the problems and provides a perspective for future applications of PET imaging in CVD studies.

## 1. Introduction

Cardiovascular diseases (CVD) are among the leading causes of death in developed countries. Every year, over 17.5 million people die from CVD, with the highest proportion recorded in middle-income and low-income regions compared to high-income regions [1,2,3]. The phenomenon is most often observed in the older age group, however, there is an increasing tendency in the economically most productive groups [4]. Over the next few years, the incidence of CVD may multiply due to the ageing of the population. The more and more common occurrence of diseases such as diabetes and obesity, as well as the conditions associated with smoking, intensify the frequency of CVD [5].

The progress of cardiovascular diseases starts from impaired endothelial function to inflammation of the vessel walls and the subsequent formation of atherosclerotic plaques, resulting in myocardial infarction and stroke. Therefore, priority is given to sensitive and non-invasive methods of early detection and characterisation of cardiovascular diseases and heart failure. Molecular imaging enables the non-invasive visualisation and quantification of biological pathways at the molecular and subcellular levels. Specially selected probes are used as a source of contrast [6]. This approach gives a better understanding and earlier lesion detection, leading to an accurate disease prognosis. One of the gold standard methods for CVD diagnostics is positron emission tomography (PET) imaging in clinical and preclinical studies. Nuclear imaging has broad clinical applications. For example, Vaz et al. extensively described medical fields of application of such imaging in, cardiology, neurology, and psychiatry [7]. The developing medical technology, innovative radiopharmaceuticals and equipment, have led to a significant increase in the number of the diagnostic and therapeutic PET procedures [8]. Its advantage over more conventional methods is to reveal dysfunctions of tissue and organ with appropriate spatial and temporal resolution in monitoring biological processes in vivo. Thanks to the selection of a radioactive isotope and modification of chemical compounds involved in biological pathways, it is possible to measure myocardial hypoperfusion, systolic and diastolic function under stress and rest conditions, and to assess the viability of the heart muscle in one scanning session.

The difficulty in CVD diagnostics results from the complexity of pathological processes and multifactorial causes of cardiac diseases. Animal models can imitate the differences in pathological conditions and give access to accurate data that is limited in clinical trials. Rodents are the most used models in preclinical research and account for approximately 95% of all animal procedures [9]. The plethora of genetically modified strains of mice and rats encourage researchers to develop new research tools for monitoring the CVD and establish new gold standard for cardiac and vessels imaging.

This review summarises the state-of-the-art and the advances in the use of markers for isotope imaging in detection of the smallest possible lesions and implementation of the therapies developed based on the use of positron emission tomography in preclinical studies on rodents. It also discusses visualisations of significant dysfunctions for a more accurate prognosis. Moreover, this review provides an assessment of the recent advances in quantitative PET tests, including the role in the detection and the correctness of the use of individual isotope markers, and the influence of the selected anaesthesia on the conducted experiments.

## 2. Principles of Molecular Imaging in Animal Model of CVD

### 2.1. PET Imaging Overview

Nuclear imaging has become a routine practice in clinical research related to diagnosis and treatment of many diseases. In nuclear medicine, radioactive isotopes are combined with other chemical compounds, enabling the construction of diagnostic or therapeutic radiopharmaceuticals also known as radioconjugates. The thus-created radioconjugates are involved in metabolic pathways, serving as a molecular probe and emitting ionising radiation outside the body. The procedures rely on the oral, intravenous, or intramuscular administration of a low dose of targeted activity. This ensures high selectivity and affinity by modification of selected particles. Scanners equipped with detection cameras measure the radiation and present the distribution of activity in the body. By designing interactive probes for individual cellular targets, researchers achieve high spatial resolution, classifying nuclear imaging as the leading technique in molecular imaging.

Imaging the disease’s progress or the treatment’s therapeutic effects using isotope monitoring is commonly used in oncology [10,11], neurology [12], and during the treatment of cardiovascular diseases [13] and infectious diseases [14]. Radioactive isotope-based imaging offers several advantages in preclinical and clinical research over magnetic resonance imaging (MRI) and computed tomography (CT), including: (i) exceptional sensitivity of nuclear techniques to the designed markers compared to the method based on Radiation detectors; (ii) possibility to monitor the concentration of the probe dynamically or statically; (iii) relative non-invasiveness of the molecular probes used for PET imaging, as both the dose and the resulting exposure of the surrounding tissues to radiation is low [15].

The growing interest in PET prompts scientists to develop newer and better molecular probes. This is possible by creating analogues, ligands of characteristic receptors, and specific antibodies, ensuring more accurate characterisation of biological phenomena. 

Like any imaging technique, it also has its limitations. PET imaging offers relatively low spatial and temporal resolution compared to techniques such as MRI and CT. The intrinsic spatial resolution of clinical PET is presumed to be in the range of 3–6 mm. The uncontrolled movements such as cardiac systolic-diastolic phases and during breathing affect the resolution, what is considered as the main PET disadvantage [16]. Compared to PET, MRI tests provide more accurate anatomical information while ensuring high resolution [17]. Intravenous injection or oral administration results in the distribution of compounds through the body before they reach their destination. Despite the trace activity of the radiocompound that has come a particular tissue, there is a low risk of irradiation in the surrounding regions [18]. The final aspect is obtaining inaccurate anatomical information using only nuclear techniques, which makes its analysis and interpretation difficult and sometimes impossible. 

Most of these difficulties can be overcome by using a multimodal approach with integrated non-invasive MRI or CT imaging techniques. Hybrid procedures are now available in preclinical and clinical practice, enabling both anatomical and physiological data on the distribution of activities to be obtained for a single test.

#### 2.1.1. PET Modalities

**Figure 1:**
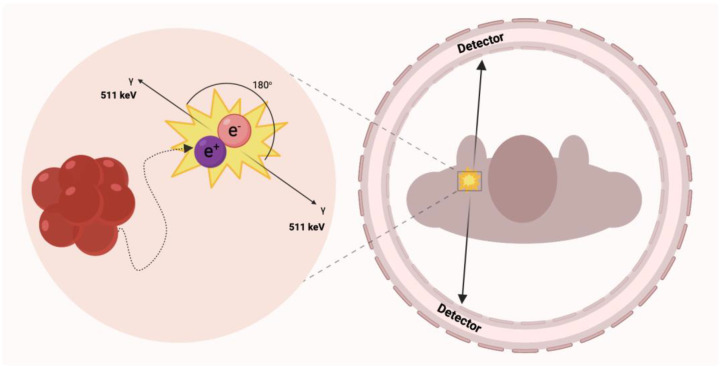
Simplified schematic of positron annihilation. At low energy, the emission of the positron particle results in the annihilation of the electron-positron pair and the production of energetic photons. Ideally, two photons are formed in a linear arrangement with an electron or positron resting energy of 511 keV.

Until now, histopathological confirmation of pathological processes was mandatory in diagnosis. Tissue biopsy is considered an invasive procedure, and it cannot be used as a common approach to identify disease progress or change. The technological evolution of non-invasive imaging techniques has made them play an essential role in evaluating the cardiovascular system functions and defining the therapeutic decision and prognosis delineation [19]. Positron emission tomography detects two coexisting photons formed by annihilation that is emitted at an angle of 180° from each other (Figure 1) [20]. Compared to single-photon emission computed tomography imaging (SPECT), in this case, only gamma rays with an energy equal to 511 keV are considered [21]. In conventional PET systems, the detector cameras comprise a series of crystal sensors and are positioned in a cylindrical gantry pattern providing an optimal field of view to handle objects of various sizes [22]. Small detectors at different angles record the coincidence of quanta in the 10 ns window from positron-emitting radioisotopes, enabling precise 3D image reconstruction and delineation of areas and volumes of interest around the target tissues.

It is worth considering the fact that many physical factors, as well as the condition of patients, affect the quality and quantitative accuracy of imaging techniques based on ionising radiation. If these factors are not adequately corrected, the further planned procedure results in a loss of quality and accuracy of tomographic images, which deteriorates significantly. One of the significant physical factors affecting image quality is photoelectric absorption and Compton scattering of high-energy annihilation photons [23]. Losing one of the annihilation quanta causes the signal loss for that coincidence. For this reason, hybrid data acquisition techniques such as PET-CT, may significantly improve the quality of the obtained images. However, the artefacts resulting from PET-CT incompatibility can lead to misinterpretation and erroneous quantification, especially when analysing small vessels in rodents. Algorithms for correcting the attenuation of image formation were developed, taking into account the attenuation of radiation by tissues adjacent to the target area. This allows for a more precise reproduction of tracer accumulation in the examined organ, which is especially desirable in perfusion studies. At the moment, in PET combined with magnetic resonance, the correction is much more complicated due to the lack of information about the attenuation of high-energy photons in MRI images. Despite the proposed approaches to overcome this drawback [24], problems remain and still need solutions in some applications.

#### 2.1.2. PET Radiocompounds

**Figure 2:**
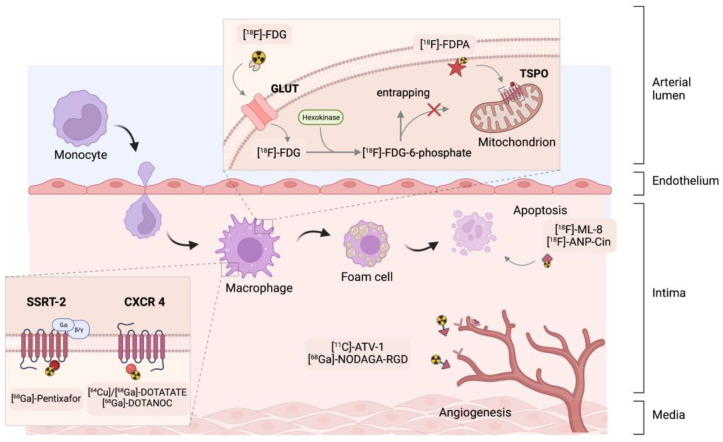
Summary of the key mechanisms of PET cardiovascular tracers. [^18^F]-ANP-Cin = [^18^F]-AlF-NOTA-PEG3-Cinnamycin, [^11^C]-ATV-1 = carbon-11-labelled pan-angiogenic receptor inhibitor, [18F]-FDG = [18F]-fluorodeoxyglucose, [^18^F]-FDPA = N,N-diethyl-2-(2-(4-(2-fluoroethoxy)phenyl)-5,7-dimethylpyrazolo [1,5-a]pyrimidin-3-yl)acetamide, [^68^Ga]-DOTATATE  =  [^68^Ga]-1,4,7,10-tetraaza -cyclododecane-1,4,7,10-tetraacetic acid-D-Phe1-Tyr3-octreotate, NOC = 1-Nal3-octreotide, RGD =  arginine-glycine-aspartic acid, NODAGA = 2-[1,4,7-Triazacyclononan-1-yl-4,7-bis(tBu-ester)]-1,5-pentanedioic acid, [18F]-ML-8  =  2-(3-[18F]fluoropropyl)-2-methyl-malonic acid, SSTR  =  somatostatin receptor sub-type 2, CXCR 4 = CXC-motif chemokine-receptor 4, GLUT = glucose transporter, TSPO  =  translocator protein.

Markers are simple or complex chemicals that can be easily traced in the study of physical, chemical or biological processes in various systems. A good marker should represent a specific component of the tested system, and possess particular features, enabling its detection and quantification, even at trace concentrations. To enable monitoring of biodistribution and retention in medical diagnostics, scientists label compounds with radioactive nuclides to form radiotracers. The use of radiotracers in PET diagnostics is based on specific phenomena that utilize the properties of the core molecule to which the radioisotope is attached. One of the most popular radiotracers is fluorodeoxyglucose ([^18^F]-FDG). [^18^F]-FDG is a glucose analog containing the isotope ^18^F, which is concentrated in cells that depend on glucose as an energy source. [^18^F]-FDG is transported across the cell membrane by proteins (GLUT 1) that facilitate glucose transport and is phosphorylated in the cell to [^18^F]-FDG-6-phosphate by the enzyme hexokinase. Once phosphorylated, it cannot exit until it is dephosphorylated by glucose-6-phosphatase. By replacing the -OH group with an ^18^F isotope the process of dephosphorylation is blocked. In consequence by measurement of the activity of ^18^F isotope we can determine the regions of body or tissue with increased [^18^F]-FDG uptake. In many cases, changes in tracer accumulation correspond to disease processes occurring in a particular region of the body. Figure 2 shows schematically the principles of action of selected radiotracers, including [^18^F]-FDG. The detailed mechanisms of action of the radiotracers discussed in this article are described in the cited literature. 

The half-life and the type of radiation emitted with specific quantum energy characterise radioactive isotopes. PET technique narrows the range of isotopes to those emitting beta plus radiation with the energy of quanta equal to 511 keV.

The most important isotopes are light nuclides of carbon, nitrogen and oxygen: ^11^C, ^13^N and ^15^O, because of the biological role of these elements in the body. This group also includes fluorine ^18^F, a stable isotope that does not occur in living organisms. The fluorine atom bonded to the carbon atom in the organic compound molecule profitably simulates some properties of the bonded -OH group and, to some extent, hydrogen and groups -CH_3_. Nevertheless, the high electronegativity of the fluorine atom can remarkably affect the physicochemical properties of the carrier molecules. Carbon-fluorine bonds show greater in vivo stability compared to carbon-hydrogen bonds. Therefore, compounds based on incorporating the fluorine isotope show greater retention and prolonged half-lives in the body [25]. Table 1 represents the principal radioactive PET isotopes list [25,26].

In clinical practice, all radiation doses are defined. Besides obtaining adequate final activity, one of the most critical elements is the purity of a radioconjugate. The radiotracers must be of high radiochemical, radionuclide, isotopic and chemical purity [27]. Radiochemical purity is explained as the ratio of the activity of its nominal chemical form to the activity of all tracer forms in radiopharmaceuticals. The cause of reduced radiochemical purity may be, for example, hydrolysis, isomerisation or decomposition of the labelled compound. For this reason, the decay products of a desirable radioconjugate, should show different chemistry to accumulate decay products in places not imaged. Rapid clearance or an easy pathway of metabolism of the products is expected, so as to limit the distribution of activities in the body and the excessive radiation load on critical organs. 

Liquid chromatography methods are widely used to purify radiopharmaceuticals in clinical and preclinical trials [28]. It is assumed that the radiochemical purity of the trace, understood as the proportion of the activity of the tag to the activity of other radionuclides in the preparation, should be 98–99%.

Almost all positron markers are obtained by bombarding a target in a cyclotron. With short-lived isotopes such as oxygen-15 or rubidium-82, a local cyclotron is essential. This generates additional high costs and makes it impossible to conduct research in individual institutions. Despite the high sensitivity of PET, the construction of compounds lasting several half-lives may require a large excess of initial activity. It is necessary to implement protective procedures for workers and lab technicians at every stage, especially with increased exposure of personnel in the initial stages of synthesis.

### 2.2. Cardiovascular Disease Modelling in Small Animal Models

In recent years, the mutual transfer of clinical and small animal model imaging techniques has provided innovative information on the pathophysiology of diseases. Non-invasive imaging methods are necessary to monitor the in vivo response to pre-set physiological or pharmacological stimuli. To achieve the best possible research results, it is necessary to observe changes at the organ, tissue, cell, gene or even the molecule level.

Knowledge of the anatomical and physiological species differences is essential for the proper understanding of preclinical results. Despite the anatomical similarities (four chamber heart), several variables in rodents compared to humans are observed. Krishnan et al. made a detailed comparison of the murine and human cardiac structures showing slight differences in the morphology of the atria and veins [29]. When imaging small animal models, the organ’s size and the high heart rate should be considered. In mice, the heart rate reaches 500–700 bpm (during isoflurane anaesthesia 250–500 bpm depending on the dose or even lower under dexmedetomidine/isoflurane or ketamine/xylazine mixture) [30,31,32]. In rats, an average of 400 BMP [33] is a standard, corresponding to 75 BMP for a healthy person. In trained individuals, the heart rate can drop to 60 BMP [34]. Additionally, rodents exhibit higher blood pressure than humans. For example, the high resting heart rate impedes the maintenance of atrial fibrillation by bringing the heart spontaneously to its original sinus rhythm. 

Difficulties in animal procedures are also caused by uncontrolled movements of the chest and other organs, as well as the surrounding lungs or the adjacent liver. The radiotracers are most often administered intravenously, hence the blood flow in large vessels may make it difficult to determine the exact outline of the area of interest. Physiological factors limit the spatial resolution to about 100 µm [35]. Gating is used to reduce motion blur for better results. Appropriate selection of scans in contraction and decompression brightens the image obtained by averaging the signal, giving useful data on the thickness of the myocardial walls and locating dysfunction. 

It is worth mentioning that studies with animals require anaesthetic drugs for PET procedures. Variable aortic pressure, diastolic pressure, right atrial pressure, and coronary perfusion pressure should be also considered. These values for rodents such as anaesthetised mice and rats are similar and were collected by Papadimitriou et al. [36]. 

The precise selection of an animal model for in vivo research should be related to the experimental set-up and depending on the disease. Inbreeding strains ensure the reproducibility and similarity of genotypes of individuals, enabling the exclusion of independent or unfavourable factors that may accompany a given physiological change in humans. Genetic modification tools provide access to multiple knock-in and knockout (KO/KI) mice that mimic spontaneous and induced CVD models. Table 2 lists the strains and how a given pathophysiological condition can be induced or monitored in rodents for in vivo cardiovascular studies [37,38,39,40,41,42,43,44,45,46].

The most commonly used models of atherosclerosis are mice deficient in apolipoprotein (ApoE −/−), whose susceptibility to the development of dyslipidemia is increased and subsequently promotes the formation of atherosclerotic plaques. It has been proven that the cholesterol level in the plasma of ApoE −/− mice are several times higher than in normal mice. Both normal and high-fat diets achieve good modelling effects by adequately simulating human lesions. Another example is mice deficient in the low-density lipoprotein receptor (LDLR −/−). When fed appropriately, can show up to ten times more plaque in plasma than wild-type mice [37]. 

The occlusion of the lumen of the artery can lead to the most fatal symptoms. Modelling of the myocardial infarction is based on permanent or temporary (with the reperfusion event) ligation of the left coronary artery. The MI procedure was induced in our group in NOD-SCID mice by ligation under isoflurane anaesthesia with 100% oxygen ventilation. This immunodeficient mouse MI model is suitable for a xenogenic cell therapy evaluation [47]. Properly planned protocols allowed us to monitor the early stages of remodelling and inflammation. Park et al. carried out a similar procedure on male Fisher rats [48]. 

Myocardial infarction models can also be used to monitor new vessel formation [49]. Cells have developed mechanisms that guarantee an adaptation to the state of hypoxia by altering the expression profile of genes related to both metabolism and angiogenesis. Radiotracers with a good pharmacokinetic profile may enable imaging of restorative angiogenesis after infarction induction or through applied gene therapies. 

Myocarditis stands as another illness example for non-invasive small animal imaging. Werner et al. used the stimulation of the body’s immune mechanisms to elicit an immune response by immunisation with porcine myosin induced in complete Freund’s adjuvant in rats [41]. Such a protocol allows the induction of autoimmune myocarditis. Moreover, Coxsackievirus B3 is the most frequent aetiological agent to induce myocarditis. Viral inflammation leads to an autoimmune response resulting in remodelling and alteration of organ function [43].

Sometimes, a different approach to inducing a disease may be considered, depending on the needs of the study. Therefore, the knowledge about the phenotype of a strain and the selection of an appropriate animal model is essential. Despite the differences in physiological and anatomical parameters between humans and rodents, mice and rats are useful research models for cardiovascular disease and give insight into the pathological mechanism of heart failure.

## 3. Radiotracer Based Imaging of Cardiovascular Related Processes, Structures, and Conditions of Heart and Cardiovascular System

### 3.1. Angiogenesis

Healing the heart tissue after a myocardial infarction is a dynamic and complex process that can be broken down into stages [50]. Angiogenesis, i.e., new vessels formation, plays a key role in the healing process. During the first stage, microvessels form from the existing vessels. This process is stimulated by the vascular endothelial growth factor (VEGF) and the fibroblast growth factors (FGF). The concentration of angiogenic factors and corresponding receptors is increased in response to an infarction. Dissoki et al. investigated VEGFR, PDGFR and Tie receptors expression in rat MI model with the carbon-11-labelled pan-angiogenic receptor inhibitor ([^11^C]-ATV-1) [51]. Studies with the rat model showed accumulation of the marker in the post-infarction zone according to the receptor activation, including the VEGF receptor, confirmed by immunohistochemical staining [52]. Radiotracers targeting VEGF are preferably used in the oncology field for tumour imaging [53]. 

Along with VEGF, integrins are also defined as important regulators of the angiogenic process [54].The integrin transmembrane receptors support cells adhesion and migration and are essential for cell survival. Moreover, integrin αvβ3 expression is not noticeable in mature vessels what makes the integrin αvβ3 is promising imaging target for angiogenesis monitoring. 

The concentration of αvβ3 increases during vasculogenesis and angiogenesis due to the action of angiogenic growth factors [55]. The discovery of the arginine-glycine-aspartic acid (RGD) peptide sequence highly selective for integrin αvβ3 was the starting point for the development of angiogenesis tracers. RGD peptides underwent chain modifications by attaching amino acids or carbohydrates. Today, there are many different synthesised RGD peptide variants.

Lang et al. had previously demonstrated the effectiveness of their cardiac induced cells (CiC) transplant in promoting angiogenesis [56]. They decided to test the effectiveness of the [^68^Ga]-NODAGA-RGD in a mouse model of myocardial infarction. It was assessed whether measuring the expression of integrin αvβ3 within the MI group would allow tracking of cardiac function with [^68^Ga]-NODAGA-RGD. MI animals showed significantly higher tracer uptake in the peri-infarct zone compared to the SHAM control group. In addition, the cilengitide inhibitor was used to stop the marker accumulation down to the level of the SHAM group. The therapeutic effects were also successfully monitored by Cai’s group by another RGD analogue [57]. Scans obtained from novel [^18^F]-AlF-NOTA-E [PEG4-c (RGDfk)] 2 ([^18^F]-alfatide II) tracer in MI Sprague Dawley rats reflected the defects identified in the [^99m^Tc]-MIBI SPECT perfusion studies.

New molecular imaging targets, such as angiomyotin (AMOT) have been reported. Angiomyotin is a protein expressed mainly in capillary endothelial cells, which is widely described in ischemic pathology [58,59]. Soluble differentiation cluster 146 (sCD146) is the endogenous ligand of the angiogenesis-promoting AMOT protein and its level reflects a new pattern in patients with heart failure [60]. 

A novel marker targeting AMOT [^68^Ga]-sCD146 has been proposed to monitor angiogenetic progress in the regeneration of myocardial tissue [61]. The group observed a significant correlation between the increasing uptake of the [^18^F]-FDG metabolic marker due to the recovery of residual perfusion, and the intensity of the [^68^Ga]-sCD146 signal. Additionally, the study compared the [^68^Ga]-RDG2 marker targeting integrin αvβ3, which showed no differences in the early imaging time. It is worth noting that the AMOT marker shows hepatic accumulation, which may affect the final evaluation of regenerative progress, but other gold standards of cardiovascular development such as [^99m^Tc]-MIBI also show a similar pattern [62].

Currently, the new vessel formation is monitored in the MI patients by myocardial perfusion. Improvement in blood flow can be considered indirect pro-angiogenic evidence, but the sensitivity of the method is limited. Flore et al. in the oncology field has described additional methods for indirect angiogenesis imaging such as hypoxia monitoring in preclinical studies [63].

### 3.2. Atherosclerosis

**Figure 3:**
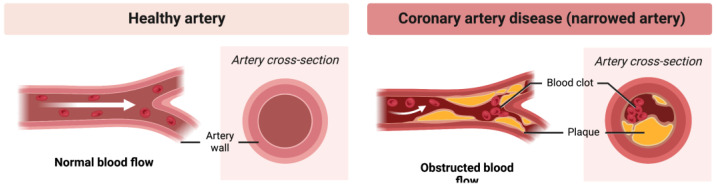
Atherosclerosis. Narrowing of the artery and obstructed blood flow.

Atherosclerosis is a chronic inflammatory disease of large arteries, which for decades remains asymptomatic and continues to dominate the list of fatal diseases. Changes in the arterial wall in the context of atherosclerosis begin in childhood, with vascular changes increasing in size with age and usually becoming symptomatic by the age of 55 [64]. Years of research have shown that the dynamics of disease development largely depend on the molecular composition and metabolic state of atherosclerotic plaques, and not on their size. The pathobiology of the disease is complex, and lamellar lesions can generally be classified as stable, leading to arterial occlusion, and unstable, with a high risk of rupture [65]. Atherosclerosis leads to the narrowing of the artery, causing obstructed blood flow (Figure 3).

Moreover, plaque rupture is responsible for 60–70% of acute coronary syndromes [62]. Hence, it is imperative to develop non-invasive tools to characterise lamina that becomes sensitive and plaques that remain stable but erode. Imaging with new probes identifying the molecular and cellular processes involved in plaque formation could provide better patient stratification and personalised strategies.

#### 3.2.1. Monitoring the Immune Cells within the Atherosclerotic Plaques

Cells such as granulocytes or macrophages in inflammatory processes accumulate in atherosclerotic plaques. Macrophages use glucose as the primary substrate, especially in anaerobic conditions in atherosclerotic plaques. Like unmodified glucose, [^18^F]-fluorodeoxyglucose ([^18^F]-FDG) is transported by the GLUT protein system and further phosphorylated to [^18^F]-FDG-6-phosphate [66]. This form results in metabolic entrapment in the cell, possibly visualising the [^18^F]-FDG accumulation in metabolically active immune cells.

Zhuang et al. performed long-term imaging of atherosclerosis development in apolipoprotein E (APoE −/−) knockout rats [38]. Imaging was performed at 12, 27 and 46 weeks of life, where from week 13, a high-fat Western diet was introduced. ApoE −/− rats developed early atherosclerotic lesions, and the calcification were detected with [^18^F]-NaF in pulmonary arteries, but the glucose-based radiotracer could not show the increased metabolic activity of immune cells in pulmonary arteries, but it was visible in the aortic arch. With Wistar rats, Shen’s group showed the accumulation of the compound in other vessels, such as the iliac artery and the abdominal aorta, after 16 and 24 weeks of feeding with a high-fat diet [39]. 

Imaging atherosclerosis with [^18^F]-FDG has potential drawbacks. For example, diabetes is a common predisposing factor to atherosclerosis [67]. Hyperglycaemia lowers [^18^F]-FDG uptake in cells, which competes with glucose for metabolic uptake. It is therefore necessary to achieve the correct glucose level in the blood before administering the marker and during the test. 

[^18^F]-FDG is non-specifically taken up by almost all tissues and organs. The analysis of the uptake of this radiotracer in arterial/venous vessels is therefore hampered by the significant spreading of the marker signal from closely adjacent structures. One example is the uptake of [^18^F]-FDG in the myocardium during PET imaging of the thoracic aorta or the coronary arteries. Moreover, pathologically increased tracer uptake in structures close to the target vessel further deteriorates the analysis of compound uptake in atherosclerotic plaques; for example, inflammation of the cervical lymph nodes near the carotid arteries. More importantly, significant uptake of [^18^F]-FDG was found in mice prone to atherosclerosis in the vicinity of calcified structures in the plaques, as confirmed by in vitro studies on human atherosclerotic arteries that showed clear binding of [^18^F]-FDG to calcification but not to other wall structures. Latinen et al. administered intravenously radiotracer to LDLR/ApoB48 atherosclerotic mice and the ex vivo accumulation in frozen aortic fragments was measured by digital autoradiography [40]. Unexpectedly, they found significant uptake near calcified plaque structures, which was more precise than non-calcified plaque structures.

Despite the widespread use of [^18^F]-FDG in the imaging of cardiovascular diseases, non-specific tracer uptake is its most significant disadvantage in the imaging of atherosclerosis. Although [^18^F]-FDG marker uptake is correlated with vascular macrophage burden, it does not necessarily differentiate morphologically unstable (inflammation) from stable (non-inflammatory) plaque. Moreover, not only is atherosclerosis characterised by macrophage-dominated inflammation, but there are many other cardiovascular diseases in which increased macrophage activity occurs without the presence of atherosclerosis. Thus, [^18^F]-FDG cannot be used to differentiate vascular atherosclerosis from diseases such as vasculitis caused by chemo- or radiotherapy or an inflammatory reaction to a foreign body such as a transplant [68,69].

#### 3.2.2. Microcalcification Imaging with [^18^F]-NaF

**Figure 4:**
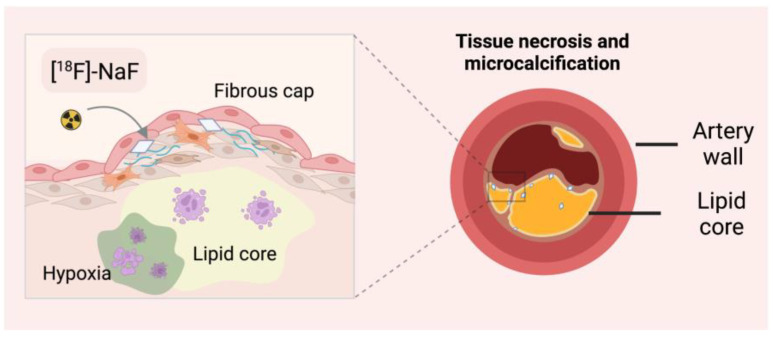
Detection of microcalcifications with [^18^F]-sodium fluoride ([^18^F]-NaF). CT–computed tomography.

Atherosclerosis is characterised by inflammation and calcification of the vessels and, consequently, their hardening. Fluoride-18 is widely used to image bone and skeletal changes in the body, but it has also been shown to be helpful in monitoring vascular mineralisation centres (Figure 4). In bones, [^18^F]-NaF diffuses into the extracellular fluid space through capillaries and is then incorporated into newly formed hydroxyapatite crystals through fluorine exchange with hydroxyl groups. Tracer uptake intensity is based on blood flow and surface availability of exposed hydroxyapatite [70]. Compared to [^18^F]-FDG, fluoride is not physiologically taken up by the heart muscle. [^18^F]-NaF has been proven specific in several independent studies to target sensitive micro-calcified plaques [71,72]. Ex vivo histological studies have shown that the resolution of [^18^F]-NaF can discriminate between micro- and macrocalcifications [70]. Importantly, CT and echocardiography cannot identify active vascular calcification, a significant factor in disease progression. The location of the calcified atherosclerotic plaques can protect the patient from sudden plaque rupture and, consequently, myocardial infarction.

Florea et al. suggest that with sodium fluoride it is possible to differentiate between the stages of plaque development [73]. They used fluoride to monitor therapy and disease progression in a mouse model of atherosclerosis. Four groups of ApoE −/− mice were fed on other diets (chow, Western diet, MK-7 supplementation and warfarin). They observed that the Warfarin group showed patchy calcifications in the CT of the proximal aorta, as confirmed by the highest [^18^F]-NaF uptake in the aortic arch and left ventricle. In contrast, the MK-7 group showed the lowest accumulation. In the group with advanced atherosclerosis, no lumps were noticed on CT, but a marker accumulation was observed, indicating the presence of microcalcification. In addition, the MK-7 group showed a similar compound uptake to the control mice group. This can provide information about the vitamin K treatment progress and the plaque’s disappearance, although these mice showed plaque on histological analysis. It was expected that the group with vitamin K supplementation would have increased uptake compared to the control group; however, the lowest accumulation compared to the other groups suggests a protective role of MK-7 against microcalcification formation.

Another conclusion is that [^18^F]-NaF has an affinity for microcalcification [74]. In groups of uraemic ApoE −/− mice exposed to chronic renal failure, non-uraemic ApoE −/− and control C57Bl/6J mice, accumulation of tracer in the aorta was observed at week 12 in the ApoE −/− groups. The differences between the groups were statistically significant. On the other hand, in the 16th week of the study, only the uraemic group showed a high uptake, while the other groups did not. The von Kossy staining quantified mineralisation showed a higher percentage of macrocalcifications in mice with no aortic uptake of the compound. The reduction of fluoride uptake in animals with positive von Kossy staining emphasises that PET imaging with [^18^F]-NaF targets the localisation of micro, not macrocalcifications, as suggested by ex vivo studies [75]. Similar results were seen in in vivo studies showing positive fluoride and PET imaging in areas where microcalcification was not detectable by CT or with small foci of macular [76]. 

Fluoride has also been shown to bind only on the surface of macrocalcium deposits [40]. It turns out that [^18^F]-NaF cannot penetrate the crystalline mass because a significant part of the hydroxyapatite is internalized [77].

It is worth bearing in mind that new calcium deposits go through the stage of microcalcification, hence continued research with the use of [^18^F]-NaF may show calcium activity, enabling the detection of new mineralisation foci [78]. [^18^F]-NaF PET imaging is limited in its relatively high cost and radiation exposure, but also [^18^F]-NaF PET imaging does not have the resolution to distinguish between medial calcification and intimal calcification [69].

#### 3.2.3. Arteriosclerosis Monitoring with Gallium-68 Based Radiocompounds

In clinical practice, fluorine-18 is commonly used to label small molecules, while gallium-68 is gaining popularity in binding with peptides, antibodies and nanoparticles [79]. The above-mentioned drawbacks and limitations of the glucose analogue have prompted the construction of new compounds with higher specificity for imaging the circulatory system of rodents. Due to distant time points, gallium-68 is effective in imaging capillaries. The strategy mainly involves targeting the membrane receptors characteristic of activated macrophages; hence imaging of active atherosclerotic plaques is directly related to monitoring inflammation.

Several markers targeting somatostatin receptors (e.g., SSTR2) have been developed, mainly for imaging lesions in oncological patients [80]. A widely studied marker for the imaging of somatostatin receptor subtype 2 (SSR-2) is a derivative of the octreotide somatostatin analogue with an attached 2,2′,2″,2‴-(1,4,7,10-Tetraazacyclododecane-1,4,7,10-tetrayl)tetraacetic acid (DOTA) chelator known as 1,4,7,10-tetraaza-cyclododecane-1,4,7,10-tetraacetic acid-D-Phe1-Tyr3-octreotate (DOTATATE). So far, the label’s efficacy has been proven to show better resolution and specificity compared to [^18^F]-FDG. In addition, no locational correlation was observed between the two markers, suggesting that binding is mediated by different cell populations [81]. The heart shows a low specific expression of SSTR2, thanks to which the metabolic signal ceases to be the main opponent in imaging arterial lesions. In rodent studies, DOTATATE confirms its effectiveness in identifying low and high-risk coronary lesions compared to [^18^F]-FDG [82]. Ex vivo measurements in the mouse aorta show increased plaque marker accumulation compared to accumulation in the vessel wall. The ratio of marker activity in the vessel to the blood pool is also taken into account. Recently, another DOTANOC analogue has been thoroughly analysed and is starting to compete with the well-known DOTATATE. DOTATATE still shows a better plaque-to-wall ratio in ex vivo studies, however, it is [^68^Ga]-DOTANOC that accumulates more in the atherosclerotic aorta. In addition, a higher aortic-to-blood pool ratio was noted [83]. 

Another way to obtain information on blood vessel health in rodents is by targeting the vascular adhesion protein-1 involved in migrating leukocytes from the blood to inflammatory sites. This protein is an endothelial adhesion molecule that contributes to the adhesion of leukocytes to the endothelium, maintaining constant expression while inflammation persists. Due to this property, vascular adhesion protein-1 becomes a promising marker in the molecular imaging of plaque inflammation. Virtanen et al. showed that immunoglobulin-like lectin 9 binding sialic acid (Siglec-9) as an adhesive protein ligand could be used as a rest for peptide labelling [84]. The [^68^Ga]-DOTA-Siglec-9 marker was created and its usefulness was compared with the modified fluorodeoxyribose ^18^F-FDR-Siglec-9. Both markers showed similar uptake results in activated atherosclerotic plaques in rodents. After intravenous administration of the tracers, dynamic PET acquisition was performed. In both cases, the inflammatory focus was visible already 10 min after administration, reaching the maximum accumulation. Additionally, the ratio of inflamed activity to blood pool was relatively high. However, ex vivo monitoring of all the exposed tissue did not show satisfactory results.

### 3.3. Myocarditis

Myocarditis is a process characterised by inflammatory lesions of the myocardium leading to myocyte necrosis [85]. Prolonged inflammation may also result in the appearance of cardiotoxic factors and the activation of immune diseases [86,87]. The consequences include impaired action potential conduction, ventricular arrhythmias and cardiomyopathy [88]. Diagnosing myocarditis is challenging due to the non-specific clinical picture. In the group of young adults, 20% of autopsies show the presence of myocarditis caused by infections. Myocarditis and cardiac sarcoidosis are the underdiagnosed causes of idiopathic cardiomyopathies [89]. Increased interest in heart inflammation research may be due to better image resolution and the emergence of the COVID-19 pandemic. Myocarditis and pericarditis may be rare adverse effects of vaccination. Recently, symptoms have been reported, e.g., in young men using multiple doses of a vaccine based on COVID-19 mRNA [90]. Studies in a mouse animal model show that this rare phenomenon can occur with accidental intravenous injection, so the recommendations for intramuscular administration are used to minimise the risk [91].

The development of animal models helps in the understanding of the cellular and molecular mechanisms of inflammatory diseases. For myocarditis, the models can be divided into two groups: infectious and non-infectious. In non-infectious models, inflammation is caused by an immune system response, while in infectious models, pathogens correspond to human myocarditis [92].

#### 3.3.1. Imaging Inflammation of the Cardiac System with [^18^F]-FDG 

MRI and [^18^F]-FDG PET examinations are used to monitor inflammation and assess cardiac abnormalities. Metabolic tests show high accuracy in detecting inflammation of the heart. Activated macrophages represent high levels of glucose transporters, resulting in rapid uptake. Serial scans were performed on rats after immunisation to determine the possibility of monitoring the progression of cardiac inflammation [41]. It is worth noting that PET imaging was performed after keeping the animals fasted for 14 h. During imaging, an increase in accumulation was noted with the highest uptake after 3 weeks. After that, a decrease was observed, compared to the perfusion marker, the accumulation of which was constant over time. This indicated an impairment of metabolic functions and therefore no perfusion damage. After 4 weeks, the heart could no longer be visually assessed.

Myocarditis is often self-limiting as can be readily demonstrated in metabolic studies with [^18^F]-FDG. According to our research, sham-operated mice show a significant increase in the accumulation of the marker in the exposed zone, which makes it necessary to plan during the study [93]. MRI studies confirmed the negative effect of intramyocardial injection on the tissue, demonstrating its dissection. 

Cellular inflammation research after MI is becoming a target mechanism in therapeutic approaches. The use of [^18^F]-FDG for imaging inflammatory processes in the heart enables the monitoring of therapeutic progress. The effect of syngeneic cardiac CiC-induced cells on myocarditis in a mouse post-infarction model was depicted [94]. Compared to other studies, ketamine and xylazine anaesthesia was used. The group demonstrated the validity of using a glucose analogue to track therapy progress, confirming the efficacy of the CiC graft in early prognostic studies to evaluate the efficacy of stem cell therapy. However, compared to studies with isoflurane as the anaesthetic agent, images with ketamine and xylazine show effective suppression of [^18^F]-FDG uptake. Cardiomyocytes and infiltrating cells show high glucose metabolism after an ischemic event, making it difficult to analyse the focal [^18^F]-FDG uptake compared to the high concentration of CD11b + monocytes/macrophages. Using ketamine/xylazine to lower serum insulin levels prevents the translocation of GLUT into cardiac cell membranes resulting in reduced uptake. This procedure avoids prolonged fasting for a glucose uptake inhibition strategy. 

Selecting the right diet during metabolic tests can significantly affect the results obtained. Following a one-week ketogenic diet, a reduction in tracer uptake by normal myocardium was observed, revealing areas of inflammation in Wistar rats [95]. In this case, the group showed that the images and results obtained are significantly better after the ketogenic diet than after the 18 h fast in both control and myocarditis animals. The detection of inflammatory lesions was highly accurate within 7 days of the diet.

#### 3.3.2. Leukocyte Monitoring in Myocarditis

Current methods of imaging active myocarditis are limited. A new marker based on the fluorine-18 isotope has been proposed-1,4,7-triazacyclononane-N, N′, N″ triacetic folic acid ([^18^F]-FOL) targeting the β folic acid receptor [96]. This receptor is expressed in activated macrophages. Myocarditis was induced in rats by immunisation with porcine myosin in complete Freund’s adjuvant. Additionally, the uptake specificity studies were supplemented by blocking by unlabeled ligand FR-β glucosamine folate. [^18^F]-FOL has been shown to show a selective uptake in activated macrophages which holds promise for the imaging of ongoing myocardial inflammation. The compound accumulated in the hearts of all animals with histologically confirmed inflammatory lesions, and no symptoms were detected in the control groups.

Another well-known compound is carbon-11-labelled methionine. L-[methyl-[^11^C]] methionine is widely used in imaging various cancers [97,98]. As a result of inflammation, the heart tissue begins infiltrating immune cells such as T lymphocytes and the previously mentioned macrophages. Using non-invasive imaging with high sensitivity can allow the target biopsy to be planned, avoiding multiple extractions. Researchers have demonstrated the utility of [^11^C]-methionine in detecting inflammatory lesions through macrophage uptake in a rat model of autoimmune myocarditis [99]. The marker revealed a high focal uptake in pathological cardiac tissues. Ten minutes after administration, the radiotracer achieves a constant level of accumulation, showing rapid uptake. However, due to hepatic metabolism, the image may be difficult to analyse due to poor contrast and blurring of the signal.

The DOTATATE shows uptake by leukocytes and polarised macrophages with the highest recorded uptake by T cells/NK cells and B cells [100]. It identifies active inflammation caused by recent MI but also can locate old ischemic injuries. The compound accumulates in the peri-infarction zone, reflecting residual macrophage-induced inflammation. Efficient regeneration depends on the presence of monocytes, which decreases 10–14 days after the ischemic event. Excessive accumulation of leukocytes may result in unfavourable remodelling of the heart and consequent organ function impairment [101]. [^64^Cu]-DOTATATE represents better resolution and results in uptake than [^68^Ga]-DOTATATE. The copper-64 isotope is characterised by a lower positron range (1 mm), leading to a better explanation of the obtained images compared to 68 Ga (4 mm) [102]. The half-life of 12.7 h makes the procedures more attractive due to the flexible test period and allows the quantitative study of the pharmacokinetics of the marker, biodistribution over time, the quantification of cardiac inflammation and cell specificity over two days [103].

Chemokines are small molecule proteins belonging to cytokines. Some of them are induced during the immune response, and responsible for pro-inflammatory processes, while others are considered homeostatic. Werner et al. studied specific CXC-motif chemokine-receptor 4 (CXCR4) ligand [^68^Ga]-Pentixafor for imaging of leukocyte infiltration. [46]. In studies carried out in murine models of transverse aortic stenosis, the radio compound uptake signal correlated with the final systolic volume seven days after surgery, and at three weeks it was inversely correlated with the LV ejection fraction [104]. The effectiveness of the marker is confirmed by the direct correspondence of the accumulation with the content of CD68 + macrophages in the myocardium. A number of leukocytes express the CXCR4 chemokine receptors; hence the obtained images and results indicate different types of immune system cells. In vivo analysis reveals an increase in the signal one week after the cardiac event indicating continued ventricular remodelling. The greater the tissue damage, the stronger the remodelling process, so determining high inflammatory activity can complement the predictive data.

#### 3.3.3. Myocarditis Imaging Based on Translocation Proteins 

Among the other available imaging methods, the one that uses translocation proteins is of interest. The translocation protein (TSPO) is an outer mitochondrial membrane protein widely used as a marker of activated immune cells in the heart and brain [105]. TSPO activates the cerebral microglia, which correlates with cardiac changes in the early and late stages of post-infarction myocarditis. [^18^F]-GE180 demonstrated the possibility of imaging early inflammation, which provides essential data in disease progression and prognosis of left ventricular remodelling [106]. One week after MI, [^18^F]-GE180 revealed increased TSPO expression localised to activated CD68+ inflammatory cells in the anterolateral infarct region. With the development of myocardial insufficiency, the marker shows an increased presence of mitochondrial proteins in distant segments. 

Recently, a new ^18^F-based ligand N,N-diethyl-2-(2-(4-(2-fluoroethoxy)phenyl)-5,7-dimethylpyrazolo [1,5-a]pyrimidin-3-yl)acetamide ([^18^F]-FDPA) has been reported showing a high affinity for TSPO and good metabolic stability. Sometimes chemical procedures using the fluorine-18 isotope cause difficulties; hence there are continuous efforts to improve the synthesis procedures. [^18^F]-FDPA with a radiochemical purity exceeding 99% was obtained in a radiochemical yield of 15 ± 3% by Keller et al. [107]. The marker was tested for in vivo biodistribution in Sprague Dawley rats. Dynamic PET imaging revealed lung and cardiac accumulation in the early acquisition phase. Relatively high initial lung activity affects cardiac outcomes; however, the heart/lung signal ratio was high. After 40–60 min, clearance from surrounding organs was noticeable. In collaboration with other entities, Mou optimised the automated synthesis of [^18^F]-FDPA [108]. In 2020, they were the first to use a radiotracer to assess myocarditis in the MI rat model. The results were compared with PET [^18^F]-FDG and [^13^N]-ammonia imaging. [^18^F]-FDPA marker accumulation corresponds to the pathological zones indicated by metabolic and perfusion studies showing excessive TSPO accumulation.

Kim et al. examined new markers in a rat model of experimental autoimmune myocarditis [109]. [^18^F]-CB251, based on imidazopyridine, and [^18^F]-fluoromethyl-PBR28 based on aryloxyanalide were proposed for evaluating inflammatory responses. [^18^F]-CB251 shows good contrast to the lungs and effectively detects inflammatory foci of the heart muscle and brain. The specificity of the tracer uptake was confirmed by in vivo blocking. The second compound showed no significant differences compared to the control groups.

[^18^F]-LW223 is also a widely described radiotracer. The uptake of [^18^F]-LW223 corresponds to TSPO expression showing increased accumulation in the zones of infarction. The studies were confirmed by ex vivo immunofluorescence staining. MacAskill assessed the kinetics of the radiotracer in the brain and heart of rats [110]. Using simplified kinetic models, they proved that a careful approach to data evaluation should go beyond single organ-focused analysis. The group was then validated using [^18^F]-LW223 to quantify macrophage-induced carditis [111].

### 3.4. Myocardial Infarction

Due to the closure of the lumen of the artery by its narrowing, pressure changes, or atherosclerotic embolism, the flow of blood is obstructed. Regional hypoperfusion and myocardial ischemia lead to a cascade of adverse events. Briefly, cardiomyocytes die, and a necrotic zone is created, resulting in impaired cardiac function and healing to non-contractile fibrosis [112]. The scar formed promotes heart failure by constantly irritating cardiomyocytes. That can be seen by the infiltrating leukocytes and the persistent inflammation. Myocardial functions stabilise as the surrounding regions catch up with the non-functional zone [92]. The necrotic zone influences the neurohormonal activation of renin-angiotensin-aldosterone and significant remodelling of the ventricles. The LV becomes confused, losing its elasticity.

#### 3.4.1. Cardiac Metabolism Imaging Using [^18^F]-Based Tracers

The parameter that can determine the decrease in functionality in the post-infarction zone is the change in the energy metabolism of the LV myocardium. As a result of an ischemic incident, the heart’s metabolism changes rapidly, impacting the proper functioning of the mitochondria [113]. Defective mitochondria inhibit the oxidation of fatty acids, carbohydrates, ketones and amino acids, and glucose by activating glycolysis [114].

The widespread availability of [^18^F]-FDG enables the implementation of cardiac metabolic studies as an appropriate and accurate complementary tool to in vivo procedures [115]. [^18^F]-FDG monitoring using a dedicated PET scanner for small animals allows for non-invasive tracking of cardiac parameters, ensuring a correlation of the size of the post-infarction zone with histological examinations. Already at the very beginning, [^18^F]-FDG surprises with its usefulness in differentiating the methods of inducing myocardial infarction [116]. Permanent LAD ligation is a frequently practised technique, but it does not transfer the preservation of the myocardium after available reperfusion surgery. The tag enables confirmation of functional data and corresponds to immunological phenomena.

Researchers examined a decline in left ventricular metabolic volume in the first days after MI. This phenomenon is possible because of remodelling and hypertrophic reaction to maintain LV stroke volume [117]. Despite the decrease in volume, there is a significant increase in glucose demand in the incident zone, with critical changes during the first fourteen days. Increased glucose uptake highlights inflammatory processes, the presence of immune cells and the high work of the heart muscle due to overload [118].

Earlier, our research group presented the possibility of using a multimodal approach in MI imaging with [^18^F]-FDG. The marker was used to evaluate inflammation after cell transplant, assess the size of the inactive zone, and monitor the progress of regenerative therapies [38]. We proposed a novel approach by creating transverse segmentation of the heart. Haemodynamic data classified animals according to the size of MI. The highest differentiation in segment uptakes was observed in the mean MI group and was confirmed by perfusion imaging. The [^18^F]-FDG imaging is used to follow the progress of regenerative therapies, both after surgical procedures such as revascularization, or the repeatedly confirmed innovative approaches using stem cells [119].

#### 3.4.2. Monitoring of Fatty Acids Metabolism 

The heart muscle requires a constant high energy supply to maintain proper contractility functions. Glucose metabolism is an essential energy source for cardiomyocytes; however, fatty acids are the primary source of acetyl-CoA in the Krebs cycle [120]. Under normal conditions, fatty acids are oxidised and degraded by β-oxidation in the mitochondria after conversion to acetyl-CoA [121]. In ischemic conditions, the dynamic balance of the glucose/fatty acid systems is disturbed. The reduced supply of glucose results in the accumulation of glycolysis products and reduced contractility of the heart cells. Mild to moderate episodes of ischemia slow down the rate of fatty acid oxidation. It is assumed that the increase in the concentration of fatty acids is a marker of myocardial infarction. However, depending on the stage of MI, the free fatty acid concentration is initially decreased and then increases, which may play an important role in the early stages [122]. The rapid reduction in oxygen availability in acute myocardial ischemia limits the oxidation in both glucose/fatty acids cycles. Due to the lower oxygen consumption, the glycolysis process becomes the heart cells’ primary energy source [123]. Hence, the importance of fatty acids in MI imaging has increased next to research on glucose metabolism by the heart muscle.

[^11^C]-palmitate was the first to be used to visualise the metabolic behaviour of cardiac fatty acids [124]. The radiocompound is a free fatty acid used to assess the enzymatic activity of carnitine-palmitoyl transferase [125]. The earliest studies showed a correlation between the size of the defect and the accumulation monitored by PET. Imaging of ^11^C is complex compared to [^18^F]-FDG because of the long-range of carbon-derived positrons compared to these from fluorine-18 [126]. Despite the well-described operation, attempts are still being made to improve the imaging method to get more accurate data. The Li group optimised the quantitative way of measuring fatty acid metabolism [127]. [^11^C]-palmitate was used to measure the exact metabolism of fatty acids, and thus: the rate of oxidation and esterification of the heart muscle, the use and uptake of fatty acids by myocardium in C57BL/6 mice in dynamic PET acquisition. However, the clinical application of [^11^C] -palmitate is limited due to its relatively difficult synthesis, the carbon-11 half-life of 20 min and the need for a local cyclotron [128]. 

In 2017, a new ^11^C-labelled u-sulfhydryl fatty acid tracer for myocardial imaging was proposed [129]. Accumulation of [^11^C]-S-methyl-16-thiopalmitic ([^11^C]-MTPA) acid in the myocardium was compared with three other compounds: [^11^C]-S-methyl-14-thiomyristic acid, [^11^C]-S-methyl-12-thiododecanoic acid, and [^11^C]-palmitate. The study was conducted in fasted mice and rats. The focus was on comparing the compound with palmitate, which shows a bi-exponential clearance from the heart, significantly complicating the data analysis. Both [^11^C] -MTPA and [^11^C] -palmitate showed high accumulation levels of both markers in the myocardium, giving an accurate picture of the organ outline. Both compounds showed rapid uptake and clearance, followed by increased activity in the cardiac blood pool. However, the [^11^C]-MTPA radiotracer proposed by Wu’s group presented the scans with better quality and higher activity against [^11^C]-palmitate. Thus, studies show that the half-life has not blocked further research on carbon-11 labelled fatty acids, and investigation into post-infarcted heart status is still ongoing.

#### 3.4.3. Monitoring Remodelling Processes of the Heart Using [^68^Ga]-Based Tracers

Activated fibroblasts’ presence greatly influences the remodelling of the heart due to their differentiation and ability to produce collagen [130,131,132]. They migrate to the peri-infarction zone, contributing to the replacement of tissues. Initial repair of fibrosis is a desirable and critical process in preventing ventricular wall rupture. Excessive fibrosis associated with persistent active fibroblasts can result in increased ventricular wall stiffness. Cardiac remodelling is a factor that gives a clear prognostic picture in patients after myocardial infarction [133]. 

One of the new assumptions in tracking the post-infarction scar spreading is the expression of the FAP activation protein in activated fibroblasts in the process of wound healing following an ischemic event [134]. Varasteh et al. investigated the possibility of imaging activated fibroblasts with the ^68^Ga-labelled FAP inhibitor ([^68^Ga]-FAPI-04) in a small animal post-infarction model [135]. They performed long-term monitoring of ligated and sham-operated Wistar rats to assess the temporal presence of activated fibroblasts. To assess the uptake and specificity of the marker accumulation, 1 week after MI, animals were administrated with a blocking dose of unlabelled FAPI-04. They observed the highest tracer uptake on day 6 in the MI area, which corresponded to the hypometabolic areas marked by [^18^F]-FDG. The tracer accumulation was significantly higher than other organs and the non-MI group. Autoradiography of cryosections and staining with hematoxylin and eosin confirmed the accumulation of the marker at the border of the post-infarction zone. 

Langer’s group examined a new radioligand [^68^Ga]-MHLL1 with a simplified synthesis procedure compared to [^68^Ga]-FAPI-based [136]. Static scans of MI mice and the blocker group were performed on days 7 and 21 after surgery. Time was based on the procedures of the previously specified time points for FAP expression [135]. The marker revealed increased uptake in the metabolically inactive MI zone and maintained through day 21. The blocking procedure significantly reduced the radioligand uptake in the entire thoracic region. However, [^68^Ga]-MHLL1 shows accumulation in the liver, which may affect the volumetric results, especially in the case of myocardial infarction with significant thinning of the ventricular wall.

#### 3.4.4. Radioisotopic Monitoring of Cardiomyocytes Apoptosis

Another symptom of myocardial infarction is cardiomyocyte apoptosis. Apoptosis is a programmed cell death in the early stages following an ischemic event [137]. Suicidal reactions may result in the continuous destruction of heart cells, ultimately affecting the size of the infarction, myocardial remodelling, and the function of the left ventricle. 

A promising small molecule probe is 2-(5-fluoropentyl)-2-methylmalonic acid (ML-10), the mechanism of which is based on incorporation and accumulation in apoptotic cells. Healthy and necrotic cells are not sensitive to it. In 2022, Fischer’s group was the first to use [^18^F]-ML-10 in PET and autoradiographic imaging for a mouse post-infarction model [138]. Short-term monitoring was performed at 2, 4, 6, 24 and 48 h after permanent LAD ligation. [^18^F]-ML-10 demonstrated the highest uptake 2 and 4 h after surgery, and then a decrease was observed, indicating a dynamic process of compound accumulation in the post-infarction area. Ma et al. decided to compare two acids [^18^F]-ML-10 and [^18^F]-ML-8 in the non-invasive detection and evaluation of apoptosis in a rat model of MI [139]. The heart-focused marker showed the highest accumulation at 120 min after administration, which was established as the optimal time point for visualising apoptosis with acids. Both [^18^F]-ML-10 and [^18^F]-ML-8 showed a myocardial focus on days 1 and 3, but were not visible on scans 5 and 7 days after surgery. The scans of both compounds overlapped the hypometabolic zone indicated by the [^18^F]-FDG study. Compared to [^18^F]-ML-10, [^18^F]-ML-8 has a lower molecular weight and may be more suitable for PET imaging. In conclusion, the group suggests that [^18^F]-ML-8 appears to be a better marker than [^18^F]-ML-10 in imaging the process of apoptosis and discriminating necrosis. Two markers [^18^F]-AlF-NOTA-PEG3-Cinnamycin ([^18^F]-ANP-Cin) and [^18^F]-AlF-NOTA-PEG3-β-Glu-RGD2 ([^18^F]-ANP-RGD2) have been proposed [140]. Sprague Dawley rats were subjected to dynamic imaging establishing a 45 min accumulation peak in the predicted apoptotic zone. The mechanism of action of the peptide cinnamycin is based on binding to the lipid phosphatidylethanolamine (PE) found in the cell membrane [141]. The main conclusions presented concern the monitoring of early-stage apoptotic cardiomyocytes using [^18^F]-ANP-Cin, while the integrin-targeting αvβ3 [^18^F]-ANP-RGD2 was noticeable in the later days of the study. The highest radiopeptide uptake was observed on the first and third days after MI, accurately indicating apoptotic cardiomyocytes in the early stages. [^18^F]-ANP-RGD2 showed good uptake in the peri-infarction zone on day seven, correlated with an increase in β3 integrin expression, and maintained the increased score until day 28 of imaging.

#### 3.4.5. Ischemic Heart Disease

Ischemia is a condition that most often affects organs such as the brain, kidneys, and heart muscles. As a result of anatomical changes that disturb the lumen of the arteries (thrombosis, embolism), general ischemia of tissues or organs occurs [142]. Ischemia leads to hypoxia, malnutrition and even necrosis of the tissues affected by the ischemic process and is responsible for the highest percentage of morbidity and mortality due to the increased risk of complications [143]. The phenomena of impaired flow are imaged inversely based on perfusion tests with rubidium-82 chloride and PET tests, or SPECT [99mTc]-MIBI imaging. The ischemic state changes the autonomic nervous system, resulting in an increased sensitivity of nerve fibres to hypoxia compared to cardiomyocytes. Myocardial infarction not only results in denervation in the area of the event, but also in the peri-infarction zone [144]. However, assessing the viability of myocardial tissue, especially in determining hibernation or revascularization changes, may require a broader approach than perfusion testing. 

Meta-[^123^I]-iodobenzylguanidine ([^123^I]-mIBG) in SPECT studies is well described, but it shows low spatial resolution. Because of the expected properties of the isotope, meta-[^18^F]-fluorobenzylguanidine [^18^F]-mFBG targeting the norepinephrine transporter NET has been proposed. The studies showed marker retention in ex vivo measurements and temporary disappearance after pharmacological treatment. The marker distribution in the rat heart depends on NET and extra-neuronal transporters with limited reuptake into the myocardium [145]. The group shows that the obtained images show high sensitivity and higher resolution than the comparable [^123^I]-mIBG, indicating an excellent quantitative correlation with the size of the post-infarction zone in the rat MI model. 

Another mIBG analogue proposed by the Sang-Keun group is meta-(3-[^18^F]-fluoropropyl) benzylguanidine ([^18^F]-mFPBG), which shows similar tissue biodistribution to [^123^I]-mIBG [146]. The validity of the use of a new analogue in imaging of the sympathetic innervation of the heart in ischemic conditions in rats was validated. All the obtained images allowed for precise monitoring of changes in the myocardial area, and the marker correlated with data from histological examinations. 

### 3.5. Radioisotopic Imaging of Perfusion

Coronary artery disease has been the leading cause of death for over 50 years, which has significantly influenced the development of diagnostic and prognostic studies of myocardial perfusion [147]. Perfusion does not have a constant value, as it depends on the organ’s need for blood flow and oxygenation, which is determined by the work intensity of the imaging system, blood pressure and heart capacity, i.e., the amount of blood that the heart pumps into the body’s blood vessels in one minute. Thus, blood perfusion through the heart muscle is a crucial marker of the proper functioning of the whole organism [148]. Dynamic PET imaging enables the monitoring of the biodistribution of radiotracers from injection to accumulation in the myocardium, which is a basic approach to quantifying blood flow. The amount of data collected from blood pool imaging determines the understanding of the strategy of cardiovascular diseases with the possibility of establishing the correct prognosis.

**Figure 5:**
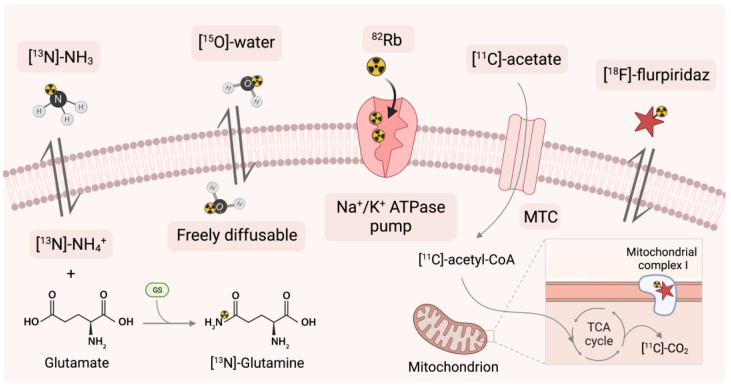
Mechanisms of action of perfusion PET tracers. [^13^N]-NH_3_ = [^13^N]-ammonia, MTC = monocarboxylate transporter, [^18^F]-flurpiridaz = 4-Chloro-5-[(4-{[2-[^18^F]fluoroethoxy]methyl}benzyl)oxy]-2-(2-methyl-2-propanyl)-3(2H)-pyridazinone, TAC = the tricarboxylic acid cycle.

The perfusion markers must be a high-performance compound with a low positron range and high image resolution. The mechanism of exemplary perfusion compounds is shown in Figure 5. A common situation is that the signal from the LV ventricle penetrates the heart muscle zone [149]. Using appropriate calculation models leads us to eliminate these inconveniences. Perfusion tests can be performed in either active or resting state. In both preclinical and clinical studies stress can be induced preferably by the use of stress-generating agents [115,150]. The most common stressors include adenosine, regadenoson and dipyridamole. Dipyridamole, however, is not approved for dynamic tests in many European countries for human use. Specific guidelines for PET/CT myocardial perfusion imaging in humans have been prepared by the European Association of Nuclear Medicine (EANM) [150]. In turn, in the studies of animal models, the use of stress-generating agents is preferred [115].

#### 3.5.1. Application of ^82^Rb as a Sodium-Potassium ATPase Pump Marker

Rubidium-82 (^82^Rb) has a similar biological pathway to that of a potassium ion (K^+^). Once in the myocardium, it is taken up as a potassium analogue in the ion exchange pump (Na^+^/K^+^ ATPase-pump) in cells [151]. Then, it is extracted proportional to blood flow: radioactivity is increased in viable myocardial cells, while the tracer is cleared rapidly from necrotic or infarcted tissue.

The first myocardial perfusion imaging study using rubidium-82 chloride in rats with experimentally induced MI was published in 2016 [152]. The paper demonstrates clear images of the myocardium. As expected, there was a loss of tracer accumulation in the post-infarction zone corresponding to the MI area in humans. Data obtained by ^82^Rb PET imaging correlate with the parameters achieved in MRI haemodynamics in assessing the LV ejection fraction and the infarct size [153]. The published images are of average quality; however, they enable marking the myocardium’s shape and the location of accumulation defects.

Pharmacological drugs such as dipyridamole or adenosine are most often used to achieve stress conditions. Research on the relationship between the markers and the selected therapy method is one of the last stages of usefulness. ^82^Rb has been tested for utility in perfusion studies in Sprague Dawley rats with certain drugs. It has been shown that drugs used in treatment do not change the radiotracer uptake, which affirms that their use does not affect the risk assessment and diagnostics in PET studies [154]. Only amiodarone, which inhibited the Na^+^/K^+^ ATPase-pump, significantly influenced the result. Since ^82^Rb enters the myocyte through a blocked pump, they do not observe tracer uptake in the myocardium.

Due to the small size of the vessels compared to the PET scans resolution, perfusion tests seemed unattainable in rodents. There are advantages to using isotopes with a short half-life like ^82^Rb. The rapid disappearance quantifies perfusion in a brief examination time of 30 min. However, the long positron range and organ size significantly limit data analysis. In a perfusion study, ^82^Rb in rats, Jensen et al. analysed image quality [155]. They applied a positron range correction based on phantom and gated rats studies. The resolution enhancement was based on the determination of% standard deviation, transfer coefficient, and recovery coefficient. This approach allowed researchers to increase spatial resolution at the expense of increased noise.

#### 3.5.2. Application of [^13^N]-Ammonia

Another radiopharmaceutical approved in clinical practice alongside ^82^Rb is [^13^N]-ammonia ([^13^N]-NH_3_) [156]. [^13^N]-NH_3_ is highly efficient in an on-site cyclotron [157]. The high first-pass ejection fraction of the myocardium, metabolism to [^13^N]-glutamine, and the imprisonment in the cardiomyocytes are responsible for the high resolution of the clinical images [158]. Following intravenous administration, it provides rapid clearance from the blood pool and uptake mainly in the heart, liver, brain, kidney and skeletal muscle. [^13^N]-NH_3_ shows a marked contrast to areas of perfusion and blood flow dysfunction [147].

In 2020, Hess et al. studied gated-[^13^N]-NH_3_ PET in C57B1/6N mice [159]. They assessed the volumetric analysis of the myocardium by combined PET, functional [^99m^Tc]-sestamibi SPECT and contrast CT analysis. Examination allowed to identify the walls of the myocardium at each cardiac gate. The anatomical images correlated with the contrast CT in estimating the ventricular volume but showed a consequently decrease. Thus [^13^N]-ammonia does not appear to be the best indicator of perfusion in rodents due to the overestimation of images [160]. On the other hand, studies in a pig model have shown that the radiotracer can be used in a single imaging session for rest and stress studies [161].

However, most PET trials are performed more frequently with rubidium-82, hence an information gap on the usefulness of [^13^N]-NH_3_ [162]. This gap is being filled by making markers discoveries and comparing the gold standards in myocardial perfusion monitoring. Recently, [^13^N]-NH_3_ is widely used in comparative studies with new perfusion markers with an optimally long half-life, such as 18F-flurpiridaz [163,164,165,166]. The conclusion is that ammonia is not the best in rodent studies compared to other newly discovered perfusion markers.

#### 3.5.3. Application of [^15^O]-Water 

Oxygen-15 labelled water ([^15^O]-water) molecule is used as a validation standard when evaluating new tracers for quantitative cardiac blood flow studies. Due to the lack of charge, it freely diffuses in the body through the membranes, quickly balancing the concentration in the blood and the heart muscle [167] and is independent of flow rate [168]. The use of a chemically and metabolically inert molecule enables a rapid differentiation of myocardial viability based on local perfusion [169]. [^15^O]-water is mainly used in clinical trials to identify coronary artery disease.

Blood flow quantification requires the determination of an input function by measuring arterial activity. In preclinical studies with small animals, frequent sampling is not feasible for obvious reasons. In addition, sampling can affect the data of the measured effect. In studies using Sprague Dawley rats, it has been proposed to intersect two measurement methods to assess input function in blood flow studies [170]. The results of femoral blood activity differed little compared to that obtained from PET images. This approach facilitates the assessment of blood flow in organs. The short half-life allows for rapid rest/stress protocols; however, [^15^O]-water exhibits a low signal-to-noise ratio [171]. This is one of the main reasons for the restriction in practice.

Herrero et al. compiled [^15^O]-water and [^11^C]-acetate and quantified myocardial blood flow in rats [172]. The obtained images allowed for the indication of the myocardial outline and the relative assessment of perfusion. Nevertheless, the determined regression slope significantly deviated from unity. The researchers conclude that blood flow measurements in the heart muscle may be underestimated by up to 26%.

#### 3.5.4. Application of [^11^C]-Acetate

The work of the heart muscle is based almost entirely on oxidative metabolism. The well-characterised [^11^C]-acetate correlating with average oxygen consumption [173] is widely described and was used to control cardiac oxidation metabolism, but now it is more often taken to perfusion studies [174]. Acetate is fully metabolised in the tricarboxylic acid cycle, also known as the Krebs cycle [175]. Energy is generated through a series of chemical reactions to oxidise the acetate. Early compound accumulation represents myocardial perfusion and oxidative metabolism. [^11^C]-acetate is rapidly converted in cells to acetyl-CoA and then oxidised to [^11^C]-CO_2_ for energy production. Persistent accumulation of [^11^C]-acetate in cardiomyocytes indicates low oxidative metabolism; conversely, a lower uptake will suggest high oxidative metabolism and release of [^11^C] in the form of [^11^C]-CO_2_. A carbon-11 half-life of 20 min necessitates immediate imaging immediately after administration. The use of labels based on this isotope are limited due to the need for an on-site cyclotron.

[^11^C]-acetate allows monitoring regenerative progress in a small animal model [176]. O’Farrell et al. decided to use [^11^C]-acetate to control the cardiotoxicity of sunitinib, which serves as a tyrosine kinase inhibitor in the treatment of solid tumours. The experiments were carried out in Balb/CJ mice and Sprague Dawley rats by administering the preparation orally for four weeks. They determined changes in the consumption of heart energy substrates during therapy. The radiotracer revealed a decrease in myocardial perfusion, indicating a reduction in myocardial oxygen consumption in individual cases by up to 80%. As monitoring continued, the parameters temporarily improved, but the results did not return to those before the treatment was started.

In comparative studies, it fared better than [^13^N]-ammonia due to the lack of uptake in the liver, which significantly affects the ease of assessing the viability of the heart, and when compiled on polar maps, it corresponded to the images obtained thanks to [^18^F]-FDG [177]. The [^13^N]-ammonia is not satisfactory with sufficient resolution in studies with mice [159], which allows ^11^C tracers.

#### 3.5.5. Application of Mitochondrial Complex-1 with Novel [^18^F]-Flurpiridaz

Properties of the fluorine-18 isotope, including the optimal half-life, make scientists create new radiotracers. The currently most studied new promising marker is [^18^F]-flurpiridaz, formerly BMS-747158 [178]. [^18^F]-flurpiridaz is a structural analogue of the insecticide pyridaben, a known inhibitor of the reduced for of nicotinamide adenine dinucleotide (NADH), which competes for a mitochondrial complex-1 of the electron transport chain binding with ubiquinone. The compound has already been tested in preclinical studies in rodents and is currently in phase III clinical trials [179]. After approximately two hours, the tracer reveals high uptake at both early and late time points. In addition, it fulfils the essential condition of good contrast by not accumulating in the surrounding organs and representing the rapid clearance of the liver. Mitochondria are abundant organelles in organs that consume a lot of energy, making them good targets for high-contrast imaging [180]. Studies in rat models of coronary obstruction showed a high correlation with tracer flow, confirmed by histopathological examinations. Recent research causes a departure from known reperfusion markers, such as [^13^N]-ammonia, due to better resolution and contrast with the background [181]. The usefulness of [^18^F]-flurpiridaz was already confirmed in stress tests and at rest in a mouse model [182]. It has been shown that the mean stress-rest ratio can be used to estimate changes in myocardial perfusion and coronary flow reserve in a small animal model.

## 4. Challenges and Future Perspectives 

Among the most commonly used compounds of all PET radiopharmaceuticals is [^18^F]-FDG [183]. It is a metabolic marker that has proven its effectiveness for many years in non-invasive imaging of metabolic changes in the brain [184], and is widely described in neoplastic lesions [185,186] but also in cardiovascular diseases [187]. [^18^F]-FDG gives a clear myocardial outline enabling quick recognition of the metabolically inactive zone. 

Many times, the [^18^F]-FDG/PET was a replacement for CT or MRI scans. By fusing images with an appropriate selection of colour coding, it made it possible to reduce the intensity of the procedures carried out with the use of animals. Disadvantages such as limited availability and the high cost of multimodal scanners are mitigated by the implementation of additional procedures with isotope-labelled glucose.

Although, reconstruction images allow for a quick assessment of the presence and size of the post-infarction zone, quantitative PET imaging using ^18^F-fluorodeoxyglucose is insufficient to assess cardiac damage after an ischemic event. Moreover, the use of [^18^F]-FDG requires additional analytical approaches, especially as active tissue remodelling processes and analysis of uptake in some segments is difficult.

The diagnosis of cardiovascular diseases is no longer solely based on the anatomical assessment of the coronary arteries, or metabolic of the cardiomyocytes, but an integrated approach to assessing both the anatomical and functional aspects of the cardiovascular system has begun. Perfusion studies can estimate imaging of vasoconstriction or microvascular pathologies associated with decreased coronary flow reserve. Assessment of the state of blood flow through the heart muscle is the basis for the prognosis of patients with CVD. The high cost of strontium-82/rubidium-82 (^82^Sr/^82^Rb) generators limits preclinical procedures. This issue can be overcome by using clinical generators. Such generators have an expiration date of 42 days, after which they still generate enough radioisotope for 5–7 weeks and can be successfully used in animal studies [153,188].

Respiratory gating is a commonly used method to obtain better resolution in heart examinations [189]. This method has also its drawbacks. The use of gating to improve resolution with a time-consuming imaging setup may result in a higher dose being used in a larger volume, which is ruled out in small animal studies. A higher dose of radiation would additionally increase the noise effects and the presence of random artefacts. Therefore, the initiation of ^82^Rb PET perfusion studies in rats is a promising start for further testing and improving our understanding of the pathophysiological uptake of ^82^RbCl in cardiac tissue and an opportunity to refine supporting techniques such as gating.

The selection of the radioisotope is also crucial. For example, for longer FAP tracking ^18^F-based radiotracers are considered to be more preferable compared to the ^68^Ga-based ones [136]. The recently developed fluorine-18-based markers targeting FAP have been tested in the rat model of radiation-induced lung damage [190] and in tumour uptake models [191,192], including the mice model of breast cancer [193].

Heart failure after myocardial infarction remains one of the leading sources of morbidity and mortality [194]. The heart, as an organ, has little ability to regenerate itself [195], but MI is followed by many processes to reduce ischemic damage. Their action is aimed at maintaining the integral structure of the heart by creating a post-infarction scar, which stabilises the ventricular wall while preserving the macro-anatomy of the heart. The goal of regenerative therapies is to rebuild the vascular path, or to replace damaged tissues with new ones with better metabolic functionality. The already existing rigid collagen zone additionally irritates the neighbouring cardiomyocytes, stimulating them to apoptosis and generating inflammation in the worst case leading to a rupture of the heart wall. As with fibroblasts and tissue replacement, excessive apoptosis plays a significant role in the prognosis of MI patients. Early identification of the stage of apoptosis and influencing the inhibition and regression of its development is a key strategy in blocking cardiomyocyte loss before transforming into a non-shrink scar [196]. As mentioned before, cardiac PET imaging can show the extinction of inflammatory processes after MI, but above all it can track the size and activity of the post-infarction zone. The evaluation of perfusion data significantly improves the diagnosis and prognosis of patients. Therefore, the use of non-invasive imaging techniques remains an extremely important in the study of damaged myocardium.

The preclinical studies of CVD carried out especially on animal models provide a number of data on benefits and drawbacks of using different radioisotope markers for diagnosis and treatment of various cardiovascular system conditions. On the one hand, isotopic techniques make it possible to obtain experimental data under the highest ethical standards related to experimental work on animals. In particular, this applies to minimizing the number of animals used by reusing a single model multiple times. On the other hand, despite obtaining multiple results like biodistribution of the compound throughout the body, affinity of radiotracers to the receptors, the precise metabolic path, the organ clearance, it should be remembered that the specific effect of the studied tracers may differ between organisms. This is especially important to consider when the clinical trials on human subjects are to be performed. Novel radioisotope markers for PET imaging of CVD still require extensive pre-clinical and clinical trial, before they can be approved for use in patients. It is important to remember that clinical trials related to the development of new cardiac markers should be conducted in accordance with guidelines provided by organizations specializing in the field of in nuclear medicine and cardiovascular imaging, such as the European Association of Nuclear Medicine (EANM) and the European Association of Cardiovascular Imaging (EACVI) [150,197]. The example of the new SYN1 tracer pointed out in the paper confirms the compatibility of the results obtained using animal models and their correlation with clinical studies [37].

## 5. Conclusions

This review provides a comprehensive analysis of the use of PET imaging of cardiovascular diseases, one of the leading causes of death worldwide. The use of different radioisotope markers and compounds labelled with ^82^Rb, ^13^N, ^15^O, ^11^C, ^18^F and other radioisotopes in preclinical procedures on rodent animal models provides unique opportunities to reflect human CVD. The use of non-invasive PET techniques with novel tracers provides high-quality images and excellent sensitivity not only in perfusion but also dynamic imaging. The latest findings in the field summarized in this review, presented alongside the still existing problems and limitations of the method provide a the perspective for future studies and possible PET applications in CVD studies.

## Figures and Tables

**Table 1:** The most common cardiac positron emission tomography (PET) radioisotopes.

Radionuclide	Half-Life (Min)	Decay	Production	Reaction	Positron Range/mm
^11^C	20.3	β^+^	Cyclotron	^10^B(d,n)^11^C	1.27
				^14^N(p,α)^11^C	
^13^N	9.97	β^+^	Cyclotron	^12^C(d,n)^13^N	
				^16^O(p,α)^13^N	1.73
				^13^C(p,n)^13^N	
^15^O	2.04	β^+^	Cyclotron	^14^N(d,n)^15^O	2.96
				^15^N(p,n)^15^O	
^18^F	110	EC, β^+^	Cyclotron	^18^O(p,n)^18^F	0.66
^64^Cu	762	EC, β^−^, β^+^	Cyclotron	^63^Cu(n,γ)^64^Cu	
				^64^Zn(n,p)^64^Cu	0.69
				^64^Ni(p,n)^64^Cu	
^68^Ga	68.3	EC, β⁺	Generator	^68^Ge/^68^Ga	3.56
			Cyclotron	^68^Zn(p,n)^68^Ga	
^82^Rb	1.27	EC, β^+^	Generator	^82^Sr/^82^Rb	7.49
^124^I	6013	EC, β^+^	Cyclotron	^124^Te(p,n)^124^I	1.70

**Table 2:** The examples of cardiovascular diseases animal models.

Medical Condition	Strain	Phenotype Occurrence or Induction	Reference
	ApoE −/− mice	display delayed lipoprotein clearance and develop dyslipoproteinemia, hypercholesterolemia and atherosclerotic lesions even when fed normal chow	[37,38]
	LDRL −/− mice	fed normal chow develop atherosclerosis gradually, can be accelerated on a high-fat diet, hyperlipidemia with human-like profile	[37,39]
	OPG −/− mice	exhibit an unexpected increase in vascular calcification in the aorta and renal arteries	[40]
	Lewis rats	immunisation with porcine cardiac myosin fraction	[41,42]
	C3H mice (Harlan)	viral myocarditis with Coxsackie virus B3 (CVB3)	[43]
Myocardial infarction and perfusion	C57BL/6J mice	coronary artery ligation	[44]
Ischemia-reperfusion	Sprague Dawley rats	development of an ischemia-reperfusion injury, temporal coronary artery ligation for 30 min followed by a 120 min reperfusion	[45,46]

## Data Availability

No new data were created or analysed in this study. Data sharing is not applicable to this article.
